# A Two-Level Model for the Analysis of Syndrome of Acute Ischemic Stroke: From Diagnostic Model to Molecular Mechanism

**DOI:** 10.1155/2013/293010

**Published:** 2013-04-04

**Authors:** Wen Dai, Xi Liu, Zhichen Zhang, Jianxin Chen, Rongjuan Guo, Hong Zheng, Xianglan Jin, Shaoxin Wen, Yibo Gao, Tiangang Li, Peng Lu, Yunling Zhang

**Affiliations:** ^1^Institute of Automation, Chinese Academy of Sciences, Beijing 100190, China; ^2^Dongfang Hospital, Beijing University of Chinese Medicine, Beijing 100029, China; ^3^Beijing University of Chinese Medicine, Beijing 100029, China

## Abstract

Prompt and accurate diagnosis of acute ischemic stroke is critical to seek acute therapy. In traditional Chinese medicine (TCM) science, there is a comprehensive system of diagnosis and medical care of acute ischemic stroke. Here we introduce a two-level model for the analysis of TCM syndrome of acute ischemic stroke. Owing to the limitation of sample size and imbalance, we focused on the analysis of wind-phlegm collateral obstruction syndrome (Feng Tan Yu Zu Zheng). Firstly, a Support-Vector-Machine- (SVM-) based diagnostic model was set up through selection of core symptoms. After pairwise undersampling, we improved the performance of prediction and generated the core symptoms-based diagnostic model of wind-phlegm collateral obstruction syndrome. Next, Pathway Pattern-based method and MetaDrug platform were used to shed light on the molecular basis of the significance of core symptoms in three complementary aspects: symptom-gene-pathway multilayer correlation network, enriched pathways, and most relevant interaction network. The integration of diagnostic model and molecular mechanism analysis creates an interesting perspective for better understanding the syndrome. The two-level model would provide a new opportunity for the study of TCM syndromes.

## 1. Introduction

Acute ischemic stroke occurs as a result of a disturbance in the blood vessels supplying blood to the brain. In spite of improvement in health care and medical condition, the prevalence of stroke has been increasing in aging society [1]. It is widely recognized that stroke is one of the leading causes of mortality and morbidity in adults and the most common cause of physical disability in many areas [2–5]. It would leave irreversible neurological impairments and cognitive decrements to the patients. In many cases, patients suffer from distressing fatigue and long-term impairment in activities such as walking and speech, which also extends to significant emotional burden like depression [1, 6]. Further, the progression of poststroke impairments might lead to a bedridden state and dementia, which generates lots of social concerns especially in the aging societies [7]. Thus, medical diagnosis and health care for acute ischemic stroke patients still present a challenge for clinicians as well as the society.

However, many acute ischemic stroke victims fail to receive medical treatment in time. In fact, stroke treatments are time sensitive [8]. Delay in reaching medical care after stroke symptom onset is the most frequent reason for low rates of thrombolytic use. Whether individuals view their symptoms as the signal of a ischemic stroke impact the delay before hospital arrival [9] and the effectiveness of urgent treatment. Indeed, prompt and accurate diagnosis of ischemic stroke is critical to seek acute therapy to reverse the problem. The diagnostic classification schemes have long incorporated two types of information: clinical observation of symptoms and radiological evidence of underlying pathophysiology [10, 11]. For example, neuroimaging and analysis of cerebrospinal fluid are widely used for diagnosis of stroke besides identification of clinical features of stroke [12].

Meanwhile, many acute ischemic stroke patients would choose traditional Chinese treatments as an alternative medical practice in China. In fact, practitioners of TCM have long focused on the medical care of stroke [13–16]. And there has been a comprehensive system of stroke diagnosis, which is characterized by its unique theoretical basis and practical experience, in TCM science. Herein, the syndromes (“ZHENG” in Chinese) of stroke are described in six aspects: wind pattern (Feng Zheng), heat pattern (Huo Re Zheng), phlegm pattern (Tan Zheng), blood stasis pattern (Xue Yu Zheng), qi deficiency pattern (Qi Xu Zheng), and yin deficiency pattern (Yin Xu Zheng) [17]. The integrated analysis of symptoms under these patterns, such as tongue, pulse, and complexion related symptoms, leads to the identification of syndromes of stroke, which helps to determine the cause, nature, and location of the illness, the patient's physical condition, and the patient's treatment [18].

 As assessing all of the recorded symptoms relevant to acute ischemic stroke in TCM science is time consuming and impractical clinically, it is pivotal to select core symptoms for the accurate diagnosis of syndromes of acute ischemic stroke. Some models have been proposed to generate diagnosis criteria for syndromes of stroke. Jiang et al. [19] developed a Bayes discriminant model for diagnosis of stroke with Qi deficiency and blood stasis syndrome. Gao et al. [20] established a diagnostic scale system for TCM syndromes of ischemic stroke. Both of these two studies obtained high diagnosis accuracy partly due to large samples (above 1000 samples), nevertheless, their symptom selection, which were based on expert experience to some extent, were not appropriate and rigorous. Kim et al. [18] applied binary logistic regression analysis with backward method on the assessed symptoms, to come up with diagnostic model for syndrome identification. The method sorted out and explained the core symptoms for each syndrome of stroke; however, it omitted the analysis of diagnosis accuracy which is the fundamental pursuit of the model. Laskowitz et al. [21] used correlation analysis to select significant features and developed a logistic model to evaluate the relationship between these features and acute stroke diagnosis. Lu et al. [22] presented a Bayesian network framework to construct a high-confidence syndrome predictor based on the optimum subset. These studies also performed well in accuracy based on selected features. In spite of the progress, few existing studies attempt to analyse the underlying mechanism of core symptoms in the molecular level, which may be responsible for their significance. Understanding the possible molecular mechanism of core symptoms would contribute to the study of syndromes. It not only helps to interpret the mechanism of action of core symptoms in the diagnosis of syndromes of stroke but also uncovers relevant biological pathways.

**Figure 1:**
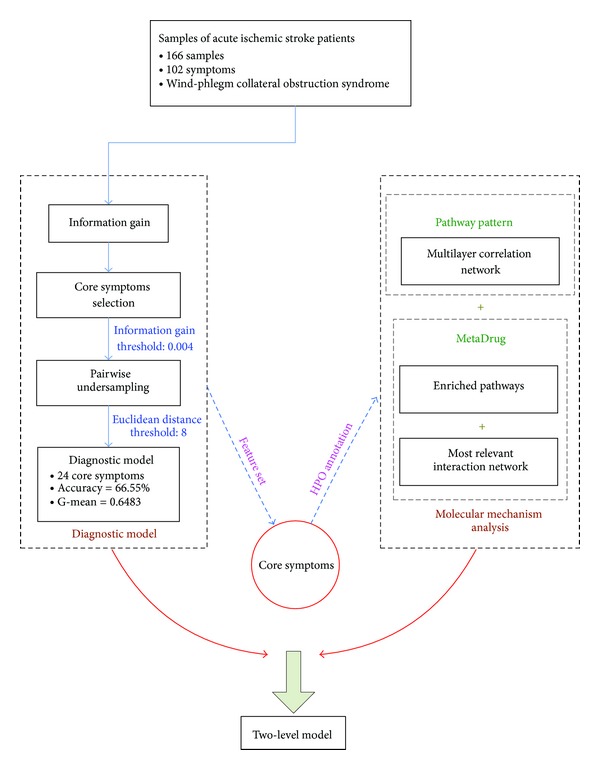
The framework of the two-level model for analysis of syndrome of acute ischemic stroke. Firstly, a diagnostic model of syndrome is generated based on selected core symptoms in the macro level. Then molecular mechanism analysis of core symptoms in the micro level is undertaken to shed light on the molecular basis of the significance of core symptoms.

 With the development of systems biology, it has been possible to integrate phenotype data, omics data, chemical data, pathway maps, and interactions of heterogeneous data in the study of molecular mechanism of core symptoms of TCM syndrome. In this work, we propose a two-level model (Figure 1) for the analysis of syndrome of acute ischemic stroke: from the macro level of diagnostic model to the micro level of molecular mechanism. SVM [23] was used to generate the diagnostic model for the syndrome of acute ischemic stroke. Meanwhile, core symptoms were chosen through organizing different groups of symptoms as the feature set of SVM model. The features that receive the best diagnostic performance are selected as the core symptoms. Next, analysis of molecular mechanism was undertaken to explore the possible way which is responsible for the significance of core symptoms. The analysis was done by two means: Pathway Pattern-based method [24] was used to build a multilayer correlation network that relates core symptoms to the mined Pathway Pattern; MetaDrug [25] was used to uncover the enriched pathways and build an interaction network to decipher the molecular basis. As the positive samples are too limited to generate diagnostic model for phlegm-heat and bowel-repletion syndrome (Tan Re Fu Shi Zheng), phlegm-heat blocking internally syndrome (Tan Re Nei Bi Zheng), phlegm-damp clouding orifices syndrome (Tan Shi Meng Shen Zheng), qi deficiency and blood stasis syndrome (Qi Xu Xue Yu Zheng), and yin-deficiency and wind-agitation syndrome (Yin Xu Feng Dong Zheng), the two-level model was suggested for the analysis of wind-phlegm collateral obstruction syndrome of acute ischemic stroke [26]. 

## 2. Methods

### 2.1. Data Preparation

We enrolled acute ischemic stroke patients within 72 hours after their ictus from several hospitals located in Northern China from August 2007 to December 2009. All the patients are aged between 35 and 75 without some specific predefined diseases. The diagnoses of syndromes were made by experienced doctors of TCM on the basis of [17]. A questionnaire of symptoms was formulated after the literature research and expert investigation. And the symptoms were observed by nerve physicians, who had been specially trained for this program, through observation, listening, interrogation, and pulse-taking along with the medical history of patients. In total, we collected 166 acute ischemic stroke samples of patients with 102 records of symptoms. Syndromes and symptoms present in the form of binary values. We omitted phlegm-heat and bowel-repletion syndrome, phlegm-heat blocking internally syndrome, phlegm-damp clouding orifices syndrome, qi deficiency and blood stasis syndrome, and yin-deficiency and wind-agitation syndrome as the number of positive samples is too limited to generate diagnostic model. Thus, we focused on the analysis of wind-phlegm collateral obstruction syndrome of acute ischemic stroke.

### 2.2. SVM-Based Diagnostic Model

We choose SVM to generate the diagnostic model, which is essentially a classification schema, because SVM is based on strong theoretical foundations and performs well in many application domains [27]. As the SVM training environment, we selected the widely used libsvm software package [28]. We used a RBF kernel function for all the SVM classifiers to prevent the choice of kernel function from affecting our results. 

As illustrated in Table 1, the dataset of wind-phlegm collateral obstruction syndrome is a small and imbalanced dataset with relatively high dimension. With imbalanced data, classifiers would judge almost all instances as the majority class because classifiers are designed to generalize from sample data and output the simplest hypothesis that best fits the data [29]. Thus, the regular SVM-based classification scheme would determine almost all the new patients as positive instances in practice. Here, we take two steps to improve the performance of SVM-based classification scheme. Firstly, we select core symptoms from the original 102 symptoms. Then samples of the majority class are combined to address the imbalance of dataset.

#### 2.2.1. Core Symptoms Selection

The irrelevant and redundant information in the original features would degrade the performance of learning algorithms. Feature selection is needed to extract core symptoms and remove secondary symptoms. In general, a feature is good if it is relevant to the target concept. Here information gain [30] is used to measure the relevance of symptoms to wind-phlegm collateral obstruction syndrome. Given a random variable *C*, we define *entropy* to measure the uncertainty of *C* as

(1) $$\begin{array}{c} H\left(C\right)=-\sum_{i}P\left({\text{c}}_{i}\right)\text{lo}{\text{g}}_{2}\left(P\left(c_{i}\right)\right), \end{array}$$

and the entropy of *C* after observing values of another variable *X* is defined as

(2) $$\begin{array}{c} H\left(C\;|\;X\right)=-\sum_{j}P\left({\text{x}}_{j}\right)\sum_{i}P\left(c_{i}\;|\;x_{j}\right)\text{lo}{\text{g}}_{2}\left(P\left(c_{i}\;|\;x_{j}\right)\right), \end{array}$$

where *P*(*c*_*i*_) is the prior probabilities for all values of *C*, and *P*(*c*_*i*_ | *x*_*j*_) is the posterior probabilities of *C* given the values of *X*. Then the information gain for *C* and *X* is given later to reflect the additional information about *C* provided by *X*:

(3) $$\begin{array}{c} IG\left(C\;|\;X\right)=H\left(C\right)-H\left(C\;|\;X\right). \end{array}$$

Here we choose syndrome as the random variable *C* and symptom as *X*. Thus the information gain between each symptom and the syndrome can be calculated to rank the significance of symptoms.

 To extract the core symptoms, different groups of symptoms, whose information gains are above a chosen threshold, are used to train the SVM-based classification scheme. As classifiers can obtain a high accuracy in imbalanced cases which is meaningless, we randomly choose ten positive samples and ten negative samples to constitute the test set, while the rest 110 positive samples and 36 negative samples constitute the training set. For each group of symptoms, the experiment is repeated for 100 times and an average accuracy of prediction in test set is calculated. Finally, the group of symptoms that obtain the highest accuracy is regarded as the core symptoms.

#### 2.2.2. Pairwise Undersampling

SVM would be ineffective in determining the class boundary and produce suboptimal classification models when the training instances are imbalanced. And the performance would drop significantly accordingly. Given a set of labelled instances *X*_train_ = {*x*_*i*_, *y*_*i*_}_*i*=1_^*n*^ and a kernel function *K*, SVM finds the optimal *α*_*i*_ for each *x*_*i*_ to maximize the margin between the hyperplane and the closet instances to it. The class prediction function for a new test instance *x* is formulated as

(4) $$\begin{array}{c} \mathrm{sign}\left(f\left(x\right)=\sum_{i=1}^{n}y_{i}\alpha_{i}K\left(x,x_{i}\right)+b\right). \end{array}$$

The instances having nonzero *α*_*i*_ values are called support vectors. In the case of wind-phlegm collateral obstruction syndrome, the positive support vectors outnumber the negative support vectors as a result of the imbalance of the dataset. According to the prediction function, the diagnosis of a new patient is likely to be dominated by positive support vectors and produce a positive prediction.

Here we introduce a strategy of pairwise undersampling to overcome the imbalance. In general, two samples which are similar to each other in core symptoms are merged into one. Given two samples *X* and *Y*, Euclidean distance is used to define the similarity between them as later:

(5) $$\begin{array}{c} \mathrm{dist}={||X-Y||}^{1/2}. \end{array}$$

The Euclidean distances were computed for all possible combinations of two samples from the dataset. Then by ranking the Euclidean distances, we got to learn the rough distribution of the samples. The combination with a smaller Euclidean distance, representing a closer similarity, got a higher ranking, and combinations with the same Euclidean distance were ranked randomly. Afterwards, we traversed the ranking and merged the two samples whose Euclidean distance is below a predefined distance threshold. The new sample was generated by averaging the two samples in the combination. It should be noted that each sample can be merged for just once in case of loss of information; in other words, combinations cannot be used for mergence if they contain samples that have been processed already. Finally, the SVM model, generated from the training set after pairwise undersampling, was applied to classify the samples in the test set. 

During the implementation, a proper Euclidean distance threshold should be selected for pairwise undersampling. In fact, threshold on the high side would merge the samples excessively and cause loss of information, while threshold on the low side leads to deficient mergence and barely satisfactory improvement of the prediction performance. Thus, we set different distance thresholds to generate several SVM models. For each Euclidean distance threshold, the experiment is repeated for 100 times, and an average accuracy of prediction in the test set is calculated. The SVM model that obtains the highest accuracy is selected.

 To measure the performance of SVM-based classification scheme, we introduce the metric of G-mean [31], which is defined as

(6) $$\begin{array}{c} g=\sqrt{\text{sen}\times \text{spe}}, \end{array}$$

where sen = sensitivity, spe = specificity. This metric has been used for evaluating classifiers on imbalanced datasets. We also use this metric to evaluate our model. Besides, we list the sensitivity and specificity separately to present a more detailed description of the diagnostic model.

### 2.3. Molecular Mechanism Analysis

Apart from generating diagnostic model for wind-phlegm collateral obstruction syndrome based on the core symptoms, we also attempt to shed light on the molecular basis of the significance of core symptoms for the purpose of a better understanding of the syndrome. The analysis of molecular mechanism is undertaken by Pathway Pattern-based method [24] and MetaDrug platform [25], respectively, to correlate symptoms with Pathway Pattern, uncover enriched pathways and construct the most relevant interaction network. By referring to the Human Phenotype Ontology (HPO) [32–34], we bridged the gap between symptoms and genes. Firstly, we searched the ontology file for HPO terms which describe phenotypic abnormalities that have similar meaning with the core symptoms. For example, three HPO terms, namely, “Somnolence” (HP:0001262), “Drowsiness” (HP:0002329), and “Paroxysmal drowsiness” (HP:0002330), were found to have similar meaning with the core symptom of “drowsiness.” Then associated genes of each HPO term were retrieved from phenotype-genes association file in HPO database. Thus, the associated genes of the three HPO terms previously mentioned might be responsible for the symptom of “drowsiness.”

#### 2.3.1. Multilayer Correlation Network Relating Core Symptoms with Pathway Pattern

Pathway Pattern [24] is extracted to reflect the biological features of core symptoms. We collected HPO terms for all of the core symptoms. Then associated genes of these HPO terms were retrieved and sorted according to their number of occurrences. Some occasionally occurring genes were removed from further analysis. And as to the remaining genes, we designate them as “relevant genes” hereinafter as the molecular mechanism analysis of core symptoms is based on these genes. Next, we prepared pathway information for the relevant genes by searching the KEGG database [35], where we could search for all pathways that a specific gene is involved. This resulted in a pathway dataset in which each record of pathways corresponds to one specific gene. The pathway dataset was used to extract the Pathway Pattern, which is in the form of association rules, with data mining method described in [24].

 Then we related core symptoms with the extracted Pathway Pattern in the reverse direction. A gene is connected with an association rule of the Pathway Pattern if the gene contains all the pathways of the association rule in its related pathways. Similarly, a core symptom is connected with a gene if the gene exists in the associated genes of the HPO terms that have similar meaning with this symptom. In this way, a symptom-gene-pathway multilayer correlation network was constructed using Cytoscape [36] to discover the molecular explanation for core symptoms.

#### 2.3.2. Enriched Pathways

On the basis of the relevant genes, we uncovered enriched pathways in MetaDrug platform [25]. A *P* value is assigned to each pathway to indicate the statistical significance of the enrichment. The enriched pathways are ranked by −log⁡⁡(*P*  value).

#### 2.3.3. Most Relevant Interaction Network

The relevant genes were used as the input list for the generation of relevant biological networks in MetaDrug. The algorithm for generating the networks was chosen as Analyze Networks algorithm. The Z-score, G-score, and *P*  value [37] are three different scoring functions used to rank the small networks. The Z-score ranks the networks according to saturation with the objects from the input list of seed nodes. The G-score modifies the Z-score based on the number of Canonical Pathways used to build the network. If a network has a high G-score, it is saturated with expressed genes (from Z-score), and it contains many Canonical Pathways. The *P*  value, which is calculated using the basic formula for hypergeometric distribution, essentially represents the probability for a particular mapping of a gene list to a network to arise by chance, considering the numbers of genes in the gene list versus the number of genes in the network. In principle, all the biological networks constitute the interaction network of input genes. However, as the interaction network built from all of the gene nodes might be too large to present; here we use the the *P*  value to prioritize the biological networks and select the top ranked network as the most relevant interaction network of the input genes, which helps to decipher the molecular basis of core symptoms.

## 3. Results 

### 3.1. Core Symptoms Selection

Information gains were computed for all of the 102 symptoms on the wind-phlegm collateral obstruction syndrome. Some severely imbalanced symptoms might also obtain high information gains; nevertheless, these symptoms are likely to be valueless in classification. For example, we cannot trust a symptom whose three negative instances all lead to negative outcome because these three negative instances are too few to be convincing. In the extreme situation, these three negative instances might all be divided into the test set. Here we set a threshold of 5 to remove the severely imbalanced symptoms whose minority is less than 5. In this way, 62 symptoms were left for further analysis.  Table 2 presents the information gains for these symptoms after sorted.

**Figure 2:**
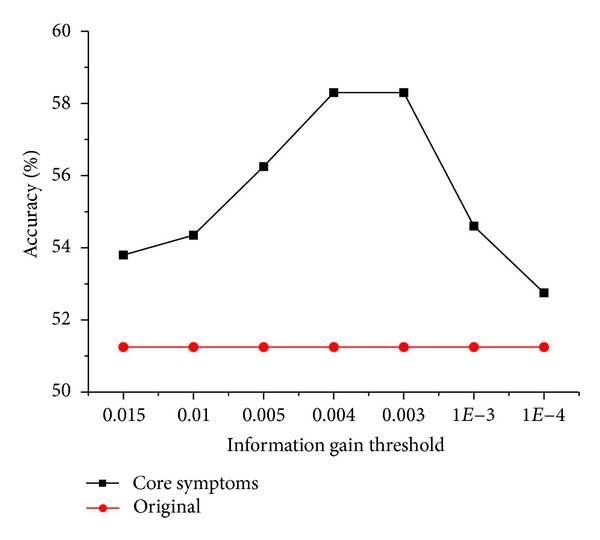
The accuracies of classification schemes based on different groups of symptoms. Symptoms whose information gains are above the threshold are selected. The black line indicates accuracies of models based on different groups of symptoms, which helps to select core symptoms. The red line presents the accuracy of the original classification scheme without feature selection as a comparison.

 Different thresholds were chosen based on the sorted information gains to select symptoms for the training of classification scheme. Some thresholds were omitted as the selected symptoms did not change much. As illustrated in Figure 2, when the threshold is set as 0.004, the classification scheme obtains the best prediction performance in test set with fewer symptoms. As a result, the 24 symptoms whose information gains are above 0.004 are selected as the core symptoms.

### 3.2. Pairwise Undersampling

**Figure 3:**
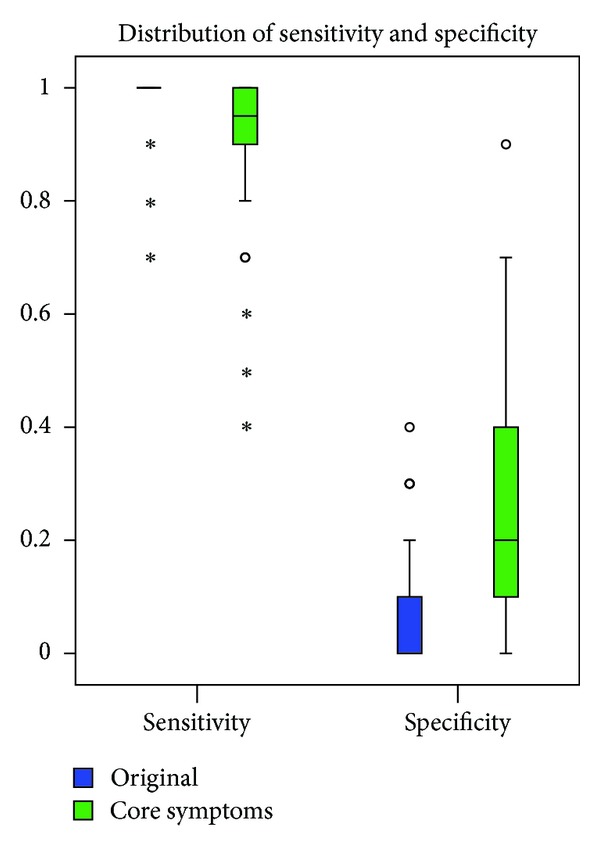
Distribution of sensitivity and specificity. Blue boxes indicate the distribution for the original classification scheme without feature selection. Green boxes indicate the distribution for the classification scheme based on core symptoms.

As illustrated in Figure 3, the original classification scheme predicts almost all the patients as the positive instances, leading to a sensitivity close to 1 and a specificity close to 0. With selection of core symptoms, the accuracy increases because of improvement in specificity. Nevertheless, the specificity is still quite low with a median of 0.2. Thus, pairwise undersampling is needed to address the imbalance and improve specificity.

**Figure 4:**
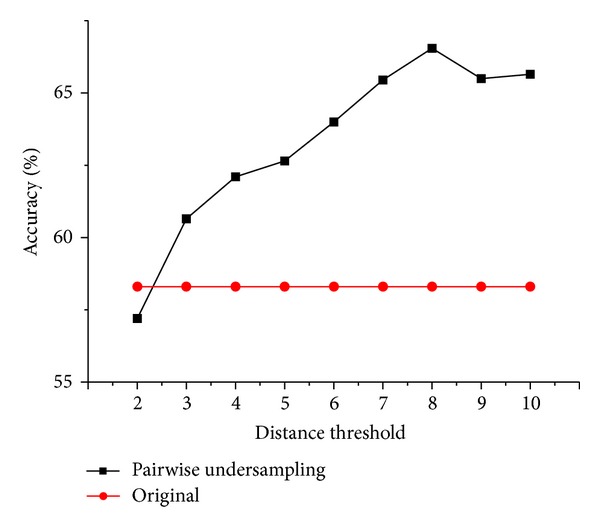
The accuracies of classification schemes after pairwise undersampling. Two samples whose Euclidean distance is below the threshold are combined. The black line indicates accuracies of models after pairwise undersampling based on different distance thresholds. The red line presents the accuracy of the original classification scheme based on core symptoms without undersampling as a comparison.

 Euclidean distances were computed based on the core symptoms for all possible combinations of two samples. Next, the Euclidean distances were ranked to describe the closeness of samples. We set different thresholds to combine two similar samples, which were then replaced by their average. As shown in Figure 4, the accuracy of classification scheme gradually increases before distance threshold = 8. Then it begins to decrease because of excessive undersampling, which leads to loss of information. When distance threshold = 8, the classification scheme obtains the highest accuracy = 66.55%. In this case, the 110 positive samples were decreased to 58 positive samples, where 52 pairs of close samples were combined and the other six samples were left alone. When distance threshold = 10, all of the 110 positive samples have been combined once, resulting in 55 positive samples left.

**Figure 5:**
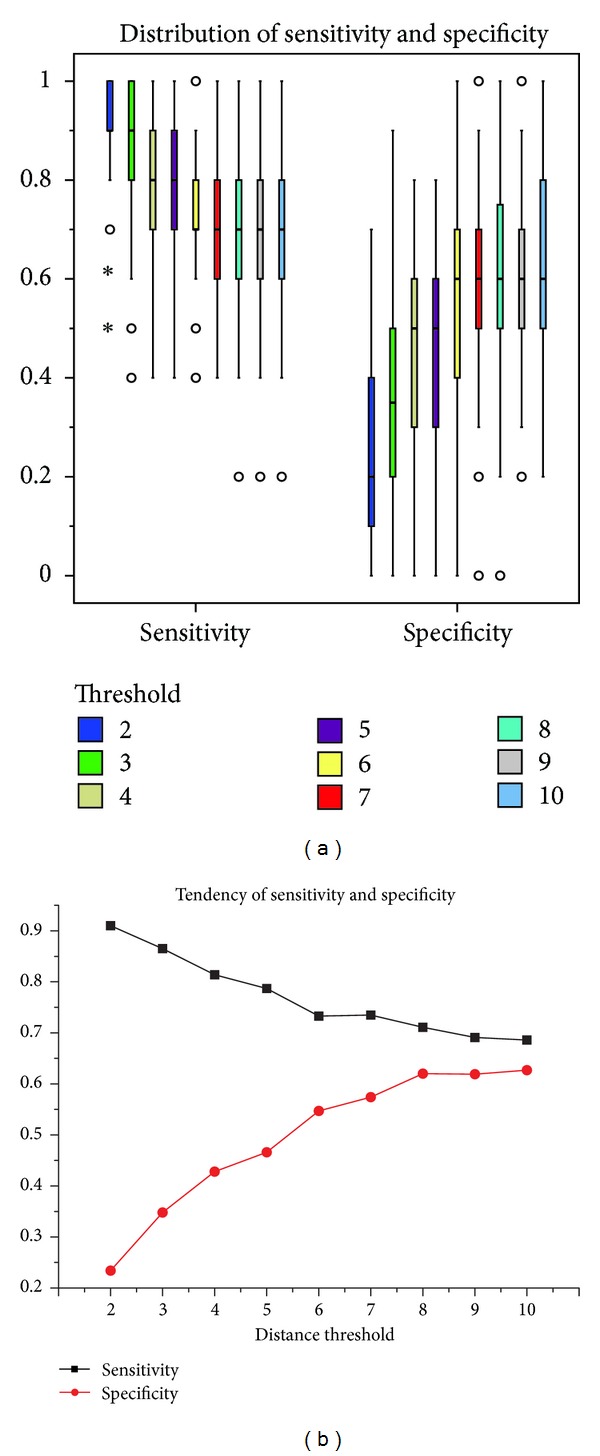
Distribution and tendency of sensitivity and specificity. (a) Boxes of different colours indicate distribution for classification schemes with different thresholds of Euclidean distances. (b) Black and red lines indicate the tendency of sensitivity and specificity, respectively, which is calculated by averaging the 100 experiments.

 To reveal the change of performances of classification schemes, the distribution and tendency of sensitivity and specificity of different models are demonstrated in Figure 5. We could find that specificity gradually increases with the undersampling of positive samples before distance threshold = 8. Although sensitivity decreases, it is insignificant compared with the improvement in specificity. After distance threshold = 8, specificity becomes steady while sensitivity keeps on decreasing.

**Figure 6:**
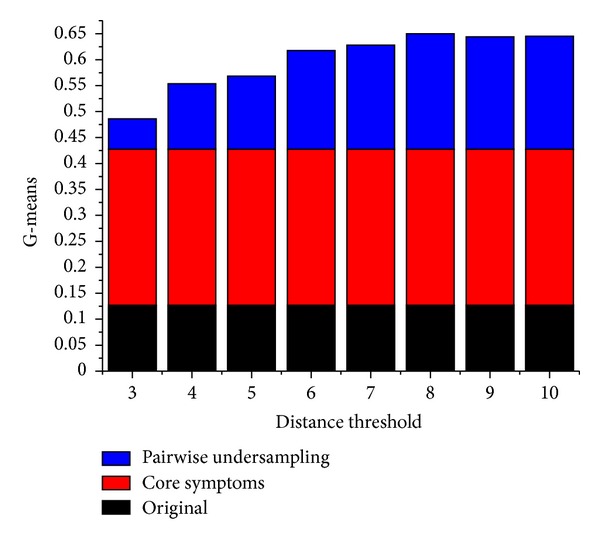
G-means for classification schemes with different distance thresholds. Black bars indicate G-mean for the original classification scheme without feature selection. Red bars indicate the rise in G-mean for classification scheme based on core symptoms. Blue bars indicate the rise in G-mean for classification schemes after pairwise undersampling.

Further, the metric of G-mean, which has been used for evaluating classifiers on imbalanced datasets, was utilized to measure the performance of SVM-based diagnostic model. As illustrated in Figure 6, the G-mean of the diagnostic model increases from 0.1253 to 0.4258 after the selection of core symptoms. Then it increases again after pairwise undersampling. When distance threshold = 8, the diagnostic model obtains the highest G-mean of 0.6483, which is acceptable considering the performance of classifiers on datasets of comparable size in [27, 29].

### 3.3. Multilayer Correlation Network Relating Core Symptoms with Pathway Pattern

We retrieved as many HPO terms as possible for each core symptom manually. For example, as to the symptom “Drowsiness”, we searched the database by keyword “drowsiness” and other synonyms like “somnolence,” and found three HPO terms which convey similar meaning: “Drowsiness” (HP:0002329), “Somnolence” (HP:0001262), and “Paroxysmal drowsiness” (HP:0002329). As shown in Table 3, most core symptoms have several corresponding HPO terms, while some core symptoms, related with pulse and fur, are not included in the database. The HPO terms were arranged to eliminate redundancy. In total, 43 different HPO terms were found.

Then associated genes of the 43 HPO terms were retrieved and sorted according to their number of occurrences. In total, 775 different genes were retrieved, of which POLG (Entrez Gene ID: 5428) occurs for 12 times and ranks the first, while 353 different genes occur only once and come last. Genes whose occurrences are below 3 are regarded as occasionally occurring genes and are removed from further analysis. As a result, 251 genes were remained and constituted the relevant genes. Next, we prepared pathway information for the relevant genes through searching the KEGG database. As some genes have no pathway information in the KEGG database, the number of records in the pathway dataset is reduced to 159. Based on the pathway dataset, the Pathway Pattern was extracted through bidirectional association rule mining (support = 0.055, confidence = 0.800) to reflect the biological features of core symptoms. As shown in Table 4, the Pathway Pattern is made up of thirteen 1-item association rules, six 2-item association rules, four 3-item association rules, and one 4-item association rule.

**Figure 7:**
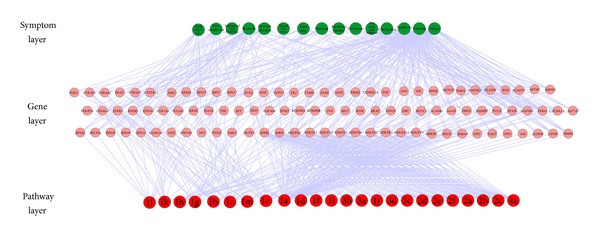
Symptom-gene-pathway multilayer correlation network. Symptom nodes are labelled with names of core symptoms. Gene nodes are labelled with gene names. Pathway nodes are labelled with symbols of the association rules of pathway pattern, which has been specified in Table 4.

In the reverse direction, we related the extracted Pathway Pattern with the 251 relevant genes and then related these genes with the 24 core symptoms. It should be noted that only complete symptom-gene-pathway interactions are maintained to correlate core symptoms with the Pathway Pattern. In other words, a node is removed if it is isolated; an edge is removed if it terminates at the gene node. In this way, a symptom-gene-pathway multilayer correlation network was formulated as Figure 7. It contains 15 symptom nodes, 98 gene nodes, and 24 pathway nodes. The correlation network helps to discover the molecular explanation for core symptoms.

### 3.4. Enriched Pathways

**Figure 8:**
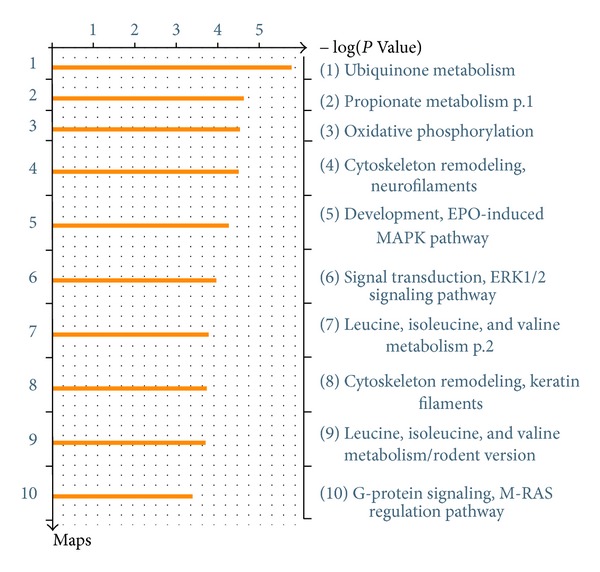
Enrichment Analysis by Pathway Maps. A *P* value is assigned to each pathway and the pathways are ranked by −log⁡(*P*   value).

**Figure 9:**
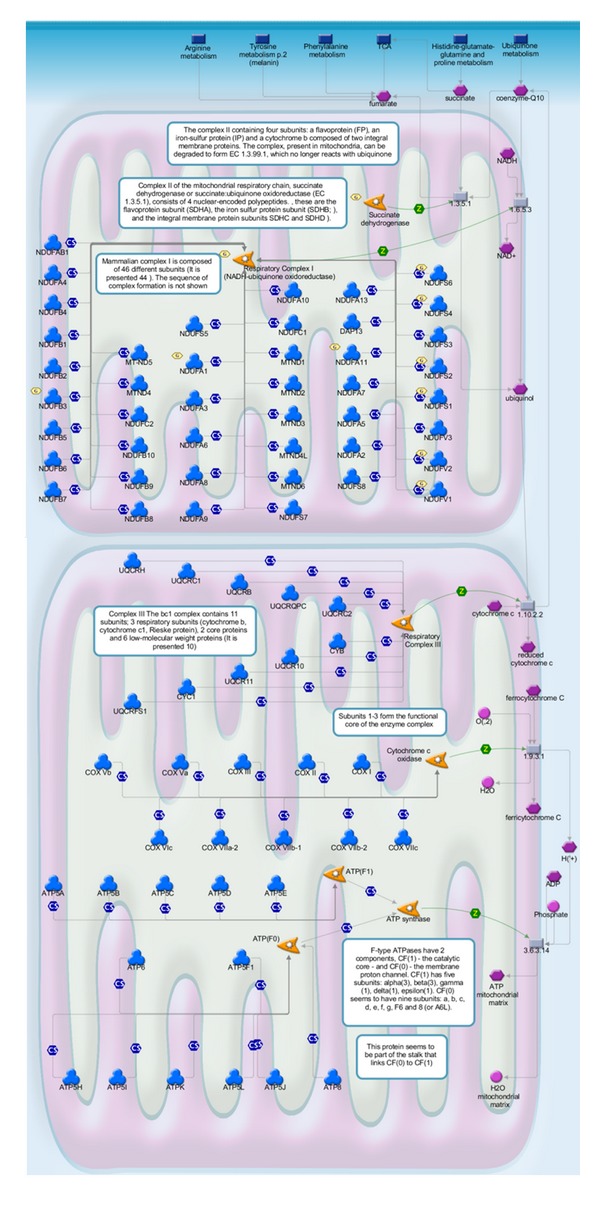
The pathway map of oxidative phosphorylation. Hexagons indicate input genes (e.g., NDUFA1, NDUFB3).

**Figure 10:**
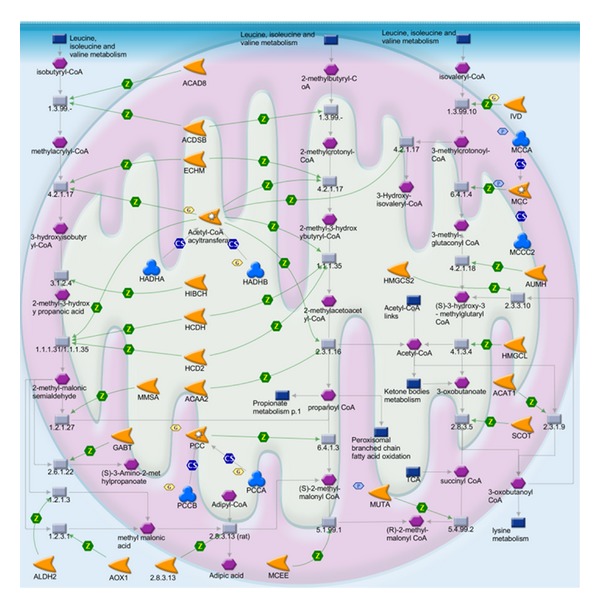
The pathway map of leucine, isoleucine, and valine metabolism. Hexagons indicate input genes (e.g., MCC, IVD).

**Figure 11:**
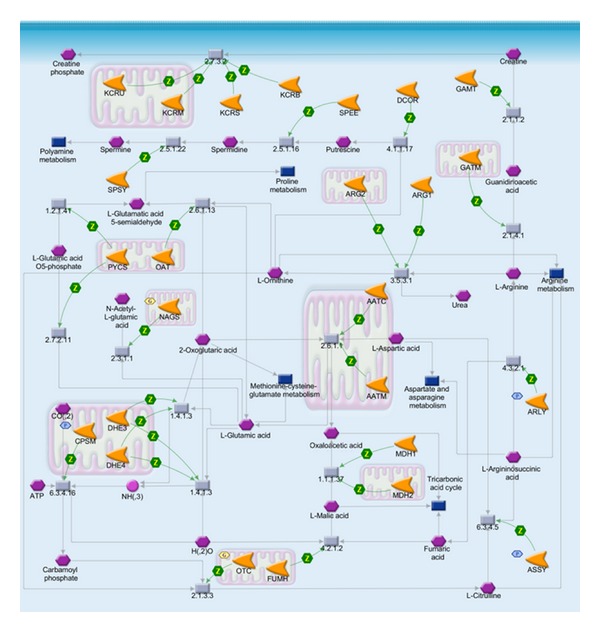
The pathway map of urea cycle. Hexagons indicate input genes (e.g., NAGS, CPSM).

The 251 relevant genes were used as the input list for the enrichment analysis by pathway maps in MetaDrug. The enriched biological pathways in Figure 8 might be responsible for the role of core symptoms in the diagnosis of syndrome of acute ischemic stroke. The relevance of some pathways has been demonstrated in previous literature. Acute oxidative phosphorylation (Figure 9) defect may have a crucial role in the pathophysiology of stroke-like episodes. The transfer RNA of leucine mtDNA mutation decreases protein synthesis and causes oxidative phosphorylation failure, leading ultimately to adenosine triphosphate depletion and energy failure [38, 39]. Low level of low-density leucine, isoleucine, and valine (Figure 10) is a characteristic of the plasma of stroke patients [40]. Besides, urea cycle disorder (Figure 11), which is among the top 20 enriched pathways, is also known to be unusual causes of stroke in some cases [41].

### 3.5. Most Relevant Interaction Network

**Figure 12:**
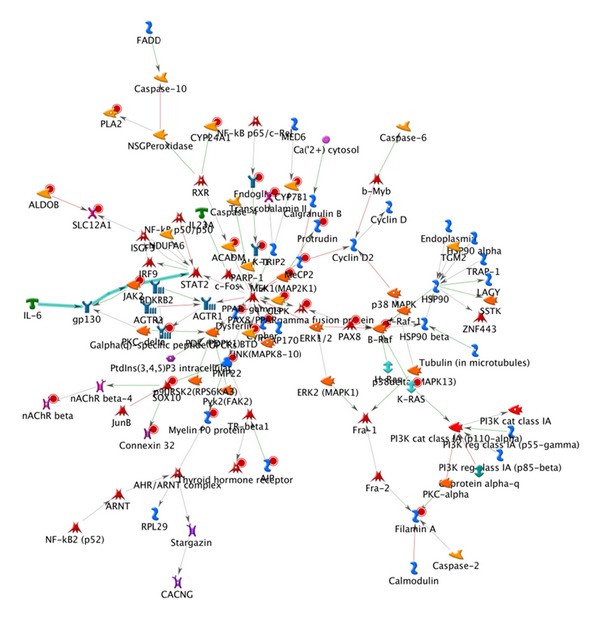
Most relevant interaction network. Thick cyan lines indicate the fragments of canonical pathways. Upregulated input genes are marked with red circles (e.g., B-Raf, PMP22).

We generated relevant biological networks in MetaDrug on the basis of the 251 relevant genes. The number of nodes in a network is set as 100. As shown in Figure 12, the most relevant biological network obtains the highest *P* value of 2.66e-37. It contains 25 input genes and one fragment of canonical pathway from IL-6 to STAT2. Some of the input genes, namely, B-Raf, ALK-1, Endoglin, PPARgamma fusion protein, and SOX 10, are significantly upregulated. The biological network helps to explore the molecular basis of core symptoms.

## 4. Discussion 

In this work, we propose a two-level model for the analysis of syndrome of acute ischemic stroke. The novel method is applied in the case of wind-phlegm collateral obstruction syndrome of ischemic stroke. The two-level model not only selects core symptoms to generate the SVM-based diagnostic model of wind-phlegm collateral obstruction syndrome but also uncovers the underlying mechanism of core symptoms in the molecular level. The molecular mechanism analysis, which contributes to the study of syndrome, is undertaken in three complementary aspects: a symptom-gene-pathway multilayer correlation network is constructed to relate core symptoms with the Pathway Pattern; enriched pathways are revealed which might be responsible for the significance of core symptoms; most relevant interaction network is generated to decipher the molecular basis of core symptoms.

 We demonstrated the framework of the two-level model by using 166 acute ischemic stroke samples of patients on wind-phlegm collateral obstruction syndrome with 102 symptoms. We showed the process of the generation of diagnostic model. To begin, information gain was computed for each symptom to decide the relevance of symptoms to the syndrome. Different information gains were then used to choose symptoms for the training of SVM-based classification schemes. The group of symptoms which yielded the best classification scheme was selected as the core symptoms. Owing to the imbalance of dataset, the classification scheme performs poorly in predicting negative instances. Through pairwise undersampling, we combined pairs of samples which are close in Euclidean space. A proper distance threshold was chosen to combine the samples in case of excessive or deficient mergence. After these procedures, we generated the SVM-based diagnostic model, which improved the accuracy and G-mean of the prediction. On the basis of core symptoms, the diagnostic model could be used to predict the wind-phlegm collateral obstruction syndrome for acute ischemic stroke patients.

 Next, we demonstrated the flow of molecular mechanism analysis to account for the significance of core symptoms. To bridge the gap between core symptoms and genes, we searched the HPO database for synonymous phenotypic terms and retrieved genes associated with these HPO terms. Then the mechanism analysis was carried out by Pathway Pattern-based method and MetaDrug platform in three complementary aspects. Pathway Pattern-based method was used to build a symptom-gene-pathway multilayer correlation network. The Pathway Pattern was extracted by mining the KEGG pathway entries of relevant genes, which are related with core symptoms. In the reverse direction, the Pathway Pattern, genes, and core symptoms were connected to generate the multilayer correlation network, which presents a new view of the relationship between symptoms and pathways. Additionally, MetaDrug platform was used to reveal the enriched pathways and construct the relevant interaction network. The enrichment analysis by pathway maps was based on the relevant genes. Some of the enriched pathways have been demonstrated to play a role in the pathophysiology of stroke in previous literature. Further the relevant genes were utilized as the seed nodes for construction of relevant biological network. The subnetwork that obtained the highest *P* value was selected as the most relevant interaction network to decipher the molecular basis of core symptoms. In combination, the analysis of molecular mechanism sheds light on the underlying mechanism for the significance of core symptoms and contributes to a better understanding of wind-phlegm collateral obstruction syndrome.

 The two-level model is a new attempt to extend the analysis of TCM syndromes. Previous research has mainly focused on building up diagnostic models for accurate prediction of TCM syndromes based on selected symptoms. The two-level model moves one step forward to analyse the mechanism of selected core symptoms in the molecular level. It combines the generation of diagnostic model with the analysis of molecular mechanism of core symptoms.

 Certainly, there are some limitations in our method. Firstly, this study takes only one syndrome of acute ischemic stroke into consideration because of the small and imbalanced dataset. This would affect the reliability and usefulness of the method. Our future work would collect more samples for a comprehensive research on the syndromes of acute ischemic stroke. Secondly, our method can suffer from annotation bias which is also the limitation of most functional annotation-based methods. Associated genes of impulse-related and fur-related symptoms need to be added in future work. Thirdly, laboratory or radiological evidence could be taken into consideration in the future in order to generate more accurate diagnostic model.

 In summary, this work demonstrates that integration of diagnostic model and molecular mechanism analysis creates an interesting perspective for better understanding of TCM syndromes. It not only makes a contribution to the research on syndrome classification but also provides insights into the molecular mechanism of the significance of core symptoms of syndrome. It would provide a new opportunity for the study of TCM syndromes.

## Figures and Tables

**Table 1:** Samples of wind-phlegm collateral obstruction syndrome.

Dataset	Positive	Negative	Total	Ratio	Features
Wind-phlegm collateral obstruction syndrome	120	46	166	2.61 : 1	102

**Table 2:** Information gains for the remaining 62 symptoms.

Symptom	Information gain
Greasy fur	0.041751
Thready pulse	0.032381
Sublingual vein bruising	0.030248
Deep pulse	0.029555
Slippery pulse	0.023376
Taut pulse	0.022498
Drowsiness	0.020716
Red tongue	0.02046
Dark purple lips	0.015375
Excessive salivation	0.014193
Dim complexion	0.013139
Heat in the palms and soles	0.013014
Headache	0.013014
Dizziness	0.010641
Fatigue	0.009921
Dry stool and oliguria	0.009029
Emotional lability	0.008032
Timid low voice	0.006736
Constipation	0.005297
Numbness	0.004979
Thick fur	0.004949
High-pitched, coarse voice	0.004901
Limb limp	0.004516
Vomiting	0.004181
Night sweat	0.003487
Red face and eyes	0.003487
Limbs twitching	0.00335
Hand, foot swelling	0.00335
Pale tongue	0.00335
Plump tongue	0.00333
Purplish tongue	0.003244
Shortness of breath	0.003108
Thin fur	0.002897
Dull expression	0.002566
Yellow fur	0.002244
Spasm of nape	0.001819
Aching and weakness	0.001818
Sweat when quite	0.001461
Wry tongue	0.001401
Poor appetite	0.00134
Dark tongue	0.001255
Fatigue and drowsiness	0.001208
Pale red tongue	0.001147
Restless	0.000649
Sticky phlegm	0.000649
Uneven pulse	0.000649
Overweight	0.000615
Vexation and irritability	0.000577
Fever	0.000412
Palpitate	0.000369
Pale complexion	0.000137
Dry stool and constipation	4.85*E* − 05
Apathy	3.93*E* − 05
White fur	1.83*E* − 05
Bitter taste in the mouth	1.72*E* − 05
Reddish yellow urine	1.17*E* − 05
Tinnitus	1.06*E* − 05
Pale tongue	1.06*E* − 05
Sweat after little movement	5.33*E* − 06
Dark purple lips and dim complexion	5.33*E* − 06
Ecchymosis on tongue	4.92*E* − 06
Predilection for cold drink	1.96*E* − 07

**Table 3:** Corresponding HPO terms for core symptoms.

Core symptom	HPO terms
Greasy fur	Smooth tongue (HP:0010298)
Thready pulse	None
Sublingual vein bruising	Abnormality of oral frenula (HP:0000190), tongue nodules (HP:0000199), glossitis (HP:0000206), and tongue telangiectasia (HP:0000227)
Deep pulse	None
Slippery pulse	None
Taut pulse	None
Drowsiness	Somnolence (HP:0001262), drowsiness (HP:0002329), and paroxysmal drowsiness (HP:0002330)
Red tongue	Tongue telangiectasia (HP:0000227)
Dark purple lips	Thick lower lip vermilion (HP:0000179), pursed lips (HP:0000205), lip telangiectasia (HP:0000214), thick upper lip vermilion (HP:0000215), and lip freckle (HP:0010798)
Excessive salivation	Abnormality of parotid gland (HP:0000197), drooling (HP:0002307), excessive salivation (HP:0003781), abnormality of the salivary glands (HP:0010286), and abnormality of salivation (HP:0100755)
Dim complexion	Pallor (HP:0000980), dull facial expression (HP:0008769)
Heat in the palms and soles	Palmoplantar hyperhidrosis (HP:0007410)
Headache	Migraine (HP:0002076) and headache (HP:0002315)
Dizziness	Vertigo (HP:0002321)
Fatigue	Syncope (HP:0001279), muscle weakness (HP:0001324), generalized muscle weakness (HP:0003324), and fatigable weakness (HP:0003473)
Dry stool and oliguria	Constipation (HP:0002019) and oliguria (HP:0100520)
Emotional lability	Emotional lability (HP:0000712) and mood swings (HP:0000720)
Timid low voice	Abnormally low-pitched voice (HP:0010300) and weak cry (HP:0001612)
Constipation	Constipation (HP:0002019)
Numbness	Impaired vibratory sensation (HP:0002495) and reduced consciousness/confusion (HP:0004372)
Thick fur	None
High-pitched, coarse voice	High-pitched, coarse voice (HP:0008377)
Limb limp	Distal muscle weakness (HP:0002460), limb muscle weakness (HP:0003690), and proximal muscle weakness (HP:0003701) and lower limb muscle weakness (HP:0007340)
Vomiting	Vomiting (HP:0002013) and nausea and vomiting (HP:0002017)

**Table 4:** Pathway pattern underlying core symptoms.

Symbol	Pathway entry	Pathway description	count
1a	hsa01100	Metabolic pathways	57
1b	hsa04151	PI3K-Akt signaling pathway	16
1c	hsa05200	Pathways in cancer	16
1d	hsa05010	Alzheimer's disease	13
1e	hsa04010	MAPK signaling pathway	13
1f	hsa05016	Huntington's disease	12
1g	hsa04510	Focal adhesion	12
1h	hsa00280	Valine, leucine, and isoleucine degradation	11
1i	hsa05012	Parkinson's disease	11
1j	hsa00190	Oxidative phosphorylation	11
1k	hsa04974	Protein digestion and absorption	10
1l	hsa03010	Ribosome	9
1m	hsa04810	Regulation of actin cytoskeleton	9

2a	hsa05012; hsa05016	Parkinson's disease and Huntington's disease	11
2b	hsa05010; hsa05012	Alzheimer's disease and Parkinson's disease	11
2c	hsa00190; hsa05010	Oxidative phosphorylation and Alzheimer's disease	11
2d	hsa00190; hsa05012	Oxidative phosphorylation and Parkinson's disease	11
2e	hsa05010; hsa05016	Alzheimer's disease and Huntington's disease	11
2f	hsa00190; hsa05016	Oxidative phosphorylation and Huntington's disease	11

3a	hsa00190; hsa05012; hsa05016	Oxidative phosphorylation, Parkinson's disease, and Huntington's disease	11
3b	hsa00190; hsa05010; hsa05016	Oxidative phosphorylation, Alzheimer's disease, and Huntington's disease	11
3c	hsa00190; hsa05010; hsa05012	Oxidative phosphorylation, Alzheimer's disease, and Parkinson's disease	11
3d	hsa05010; hsa05012; hsa05016	Alzheimer's disease, Parkinson's disease, and Huntington's disease	11

4a	hsa00190; hsa05010; hsa05012; hsa05016	Oxidative phosphorylation, Alzheimer's disease, Parkinson's disease, and Huntington's disease	11
